# Severe progressive scoliosis in an adult female possibly secondary thoracic surgery in childhood treated with scoliosis specific Schroth physiotherapy: Case presentation

**DOI:** 10.1186/s13013-016-0098-3

**Published:** 2016-10-17

**Authors:** Andrea Lebel, Victoria Ashley Lebel

**Affiliations:** 1 Ottawa & District Physiotherapy Clinic, Scoliosis Physiotherapy and Posture Centre, McLeod Street, Ottawa, K2P 0Z8 Canada; 2 Saba University School of Medicine, Saba, Dutch Caribbean Netherlands

## Abstract

**Background:**

Scoliosis is a complex three-dimensional (3D) spinal deformity. Acquired scoliosis in early childhood may progress into adulthood and pose an increased risk of health problems and reduction in quality of life. In Canada, patients with scoliosis are not referred for physiotherapeutic scoliosis-specific exercises (PSSE) despite the fact that Schroth physiotherapy, a scoliosis-specific 3D posture training and exercise program, can be effective in reducing pain and improving scoliosis curves, vital capacity, and overall quality of life in scoliosis patients. This case presentation shows that indeed adult curve progression can be stopped and even reversed with scoliosis specific Schroth physiotherapy (SSSPT) in an adult patient with scoliosis.

**Methods:**

This is a retrospective case presentation involving a 23-year-old female scoliosis patient who began an outpatient Schroth physiotherapy exercise program and was initially monitored monthly and then annually for improvement in measurements of angle of trunk rotation (ATR) and chest expansion and improvement in vital capacity measured with incentive spirometry. Photos were taken to document body image periodically throughout Schroth physiotherapy treatment. Additionally, the patient completed SRS-22 quality of life questionnaires every 2 years to evaluate daily function, pain, self-imagine, mental health, and scoliosis management satisfaction.

**Results:**

Within one month of beginning SSSPT, the patient reported no more back pain and within 2 months, reported improved breathing. The patient also benefitted from improved chest expansion, reduced scoliosis curve angles (measured in Cobb degrees), increased vital capacity, decreased ATR, and higher SRS-22 scores. She became more active and resumed all athletic activity within 8 months of beginning Schroth physiotherapy.

**Conclusions:**

Adult scoliosis patients are not routinely referred for PSSE in Canada, even though Schroth physiotherapy, a form of PSSE, is shown to be effective in this case presentation. The patient in this case presentation was successfully treated with Schroth physiotherapy. Long-term comprehensive Schroth physiotherapy, to help correct and maintain proper posture in all aspects of daily living, should be part of scoliosis management for adult scoliosis patients in Canada to stop and reverse curve progression and to improve overall quality of life.

## Background

Scoliosis is a complex three-dimensional (3D) spinal deformity [1]. In its most common form, idiopathic scoliosis (IS), there is no identifiable cause, but there is believed to be some triggering event [2] which leads to spinal curvature (measured in Cobb angles) and asymmetric loading of the spine resulting in spinal deformation. One such triggering event may be rib fusion after thoracic surgery during infancy for an unrelated medical condition. A case presentation published by Korovessis et. al. in 1993 describes a 6-year-old boy with scoliosis by acquired rib fusion after thoracotomy in early infancy [3]. Acquired scoliosis in early childhood may progress into adulthood and may pose an increased risk of health problems [4] and reduction in quality of life in later years [5, 6]. The Scoliosis Research Society (SRS) treatment guidelines for adults with scoliosis is based on pain management, core strengthening exercises, and surgery for symptom relief to improve quality of life.

In Canada, adult patients with scoliosis are not referred for physiotherapeutic scoliosis-specific exercises (PSSE) [7, 8] despite the fact that Schroth physiotherapy [9, 10], a type of PSSE involving scoliosis-specific 3D posture training and exercise programs, can be effective [11] in reducing pain and improving scoliosis curves, vital capacity, and overall quality of life in scoliosis patients [12]. This retrospective case presentation shows that indeed adult curve progression, averaging 1° per year in severe scoliosis [13, 14], can be stopped and even reversed [15] with scoliosis specific Schroth physiotherapy (SSSPT) in an adult patient with scoliosis possibly acquired by rib fusion after thoracotomy in early childhood.

### Case presentation

In 1988, a 4 cm benign ganglioneuroblastoma [16] was surgically removed from the left posterior rib cage, adjacent to the thoracic spine, of a 3-year-old girl. Post-surgical radiographs showed a straight spine. Five years later, when the patient was 8 years old, radiographs showed left 4^th^ and 5^th^ rib fusion. In 1999, at the age of 11, the patient was diagnosed with adolescent idiopathic scoliosis (AIS) by her family physician and was managed with the “wait and see” observation method at the children’s hospital in Toronto, Canada, until the age of 17. Radiographs taken between the ages of 8 and 17 were no longer available at the time of this case presentation, but the patient’s medical record did mention a diagnosis of scoliosis at age 8, without scoliosis follow-up. The patient was only monitored for recurrence of the ganglioneuroblastoma. At the age of 15 and again at the age of 17, spinal fusion surgery was recommended to the patient but the patient refused.

In November 2008, at the age of 23 years, the patient presented for an initial physiotherapy evaluation, complaining of a 2-year history of severe (7/10 on the pain scale) intermittent episodes of low back pain and shortness of breath while going up stairs. Her pain and shortness of breath had progressively worsened to the point where she had to discontinue all physical activities, including scuba diving because her rapid respiratory rate forced her to use up her oxygen supply in half the amount of time. At times, her back pain was so severe that she was unable to get out of bed.

**Fig. 1:**
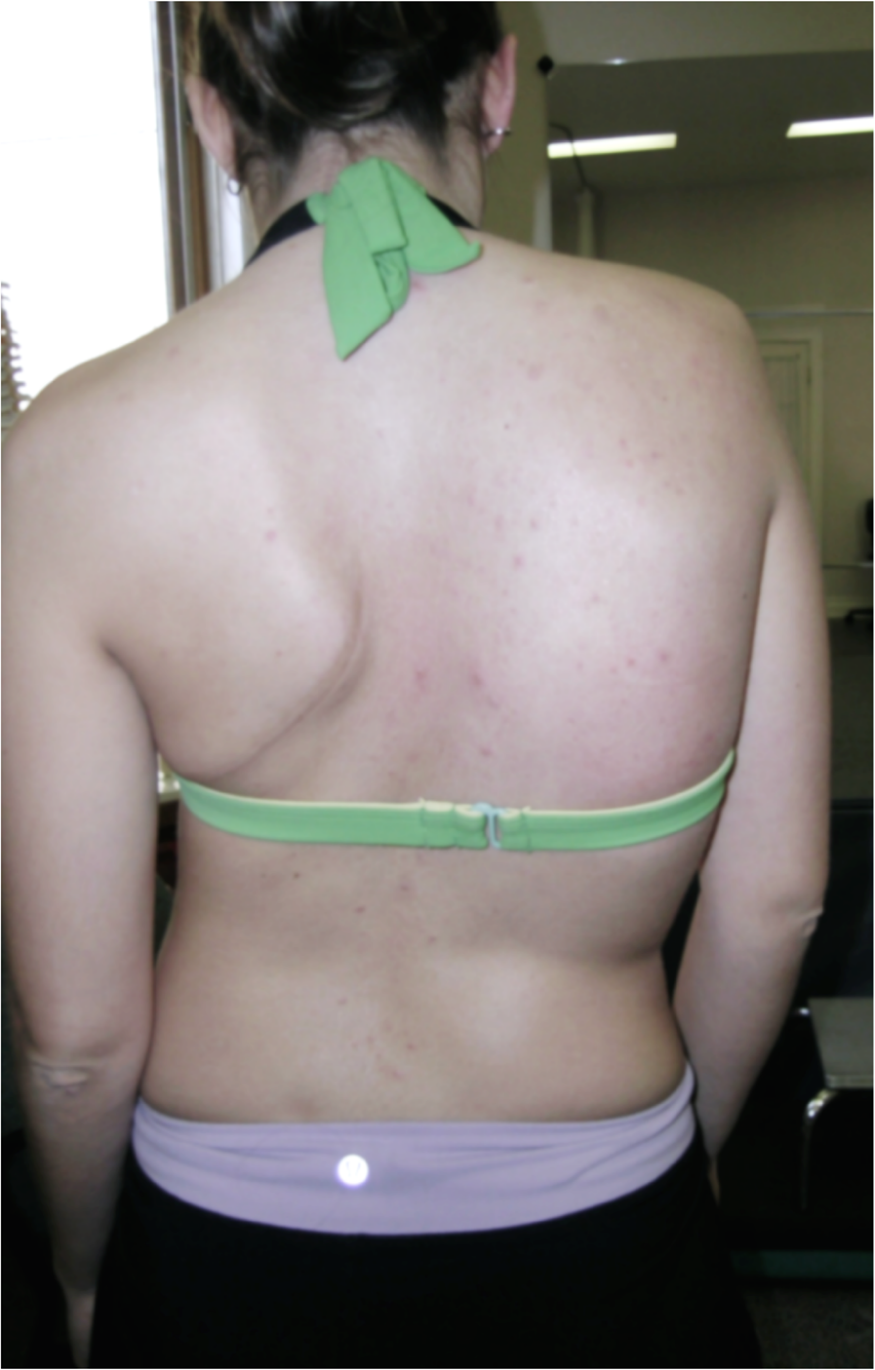
Patient photograph taken at initial physiotherapy assessment in November 2008 shows signs of severe scoliosis. Signs of severe scoliosis are evident: thoracic convex right side shoulder elevation, thoracic convex right side ribcage protrusion resulting in a rib hump, and thoracic concave left side shoulder depression and retraction

**Fig. 2:**
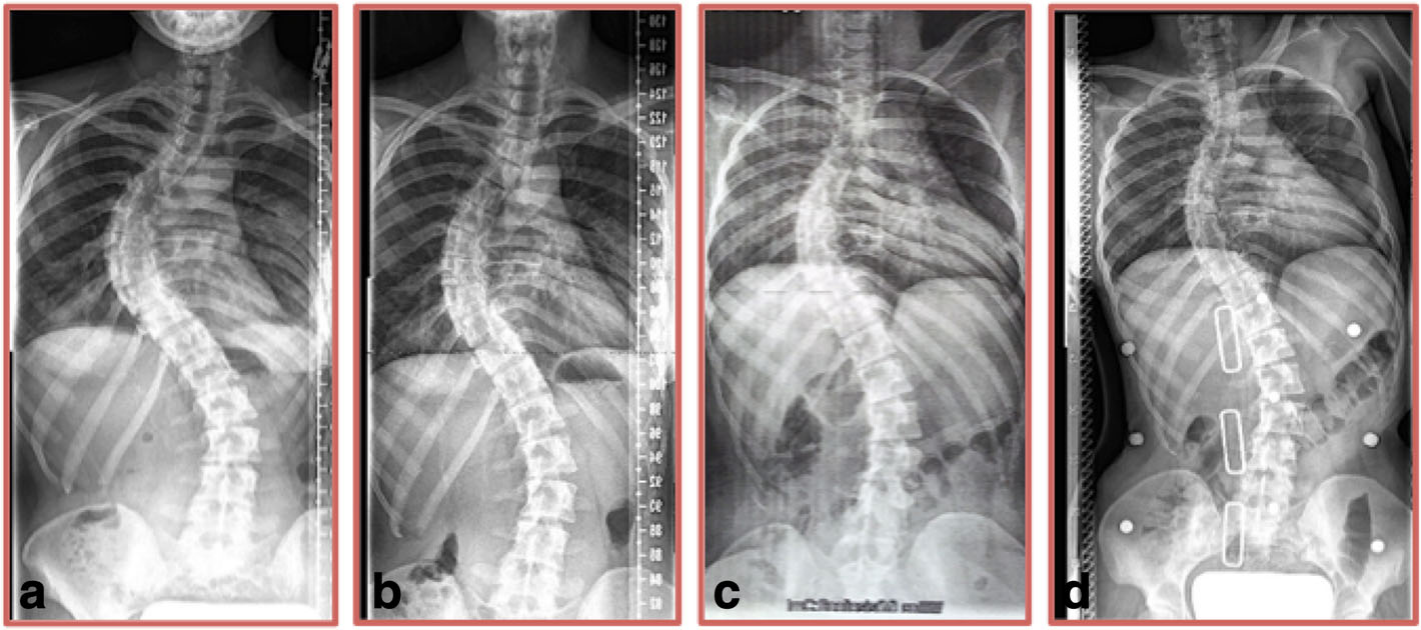
Case presentation patient radiographs 2008–2009. **a** Radiograph taken in November 2008 shortly after initial physiotherapy assessment showing a 70-degree Cobb angle thoracic curve and a 48-degree Cobb angle lumbar curve. **b** Radiograph taken in July 2009, 9 months after beginning Schroth physiotherapy, showing reduced thoracic and lumbar Cobb angles of 58 and 43°, respectively. **c** Private radiograph taken in July 2009 during a Schroth exercise showing decreased thoracic and lumbar Cobb angles of 48 and 33°, respectively. **d** In-brace radiograph taken in November 2009 showing even further reductions in scoliosis curves to 38° thoracic Cobb angle and to 30° lumbar Cobb angle

A prior radiograph from 2006 showed a 68-degree Cobb angle thoracic curve and a 47-degree Cobb angle lumbar curve. Upon initial physiotherapy assessment, there was visible severe spinal deformity (Fig. 1). The thoracic angle of trunk rotation (ATR) was 19° and chest expansion was 1.5 cm at the nipple line. Chest auscultation revealed absent breath sounds over the lower lobe of the left lung. Inspection of the chest wall showed a 10 cm long scar resembling a lobectomy. Concern for more serious underlying lung pathology warranted that repeat radiographs be obtained. The radiographs taken in November 2008 shortly after initial physiotherapy assessment revealed a 70-degree Cobb angle thoracic curve and a 48-degree Cobb angle lumbar curve (Fig. 2a), reflective of the average 1° annual scoliosis curve progression observed in adults. In addition to curve progression, the follow-up radiograph showed fused 4th and 5th left ribs, a new finding for the patient at the time. In December 2008, the patient began Schroth physiotherapy, focusing on directional breathing and scoliosis-specific exercises for a 3C curve type [17] (according to Schroth scoliosis curve classification).

## Methods

This is a retrospective case presentation involving a 23-year-old female scoliosis patient. In December 2008, the patient began 1-h Schroth physiotherapy clinic sessions 2–4 times a month in combination with a 45–60 min home exercise program (HEP) 3–5 times a week. The patient was initially monitored monthly and then annually for improvement using measurements of ATR [18], chest expansion [19], and vital capacity [20]. Photos were taken to document body image [21] periodically throughout Schroth physiotherapy treatment. Additionally, the patient completed SRS-22 [22] quality of life questionnaires (score range 0–5) in 2011, 2013, and 2015 to evaluate daily function, pain, self-imagine, mental health, and scoliosis management satisfaction. Signed consent was obtained directly from the case presentation patient for treatment, photos, data collection, and publication of this case presentation.

## Results

**Fig. 3:**
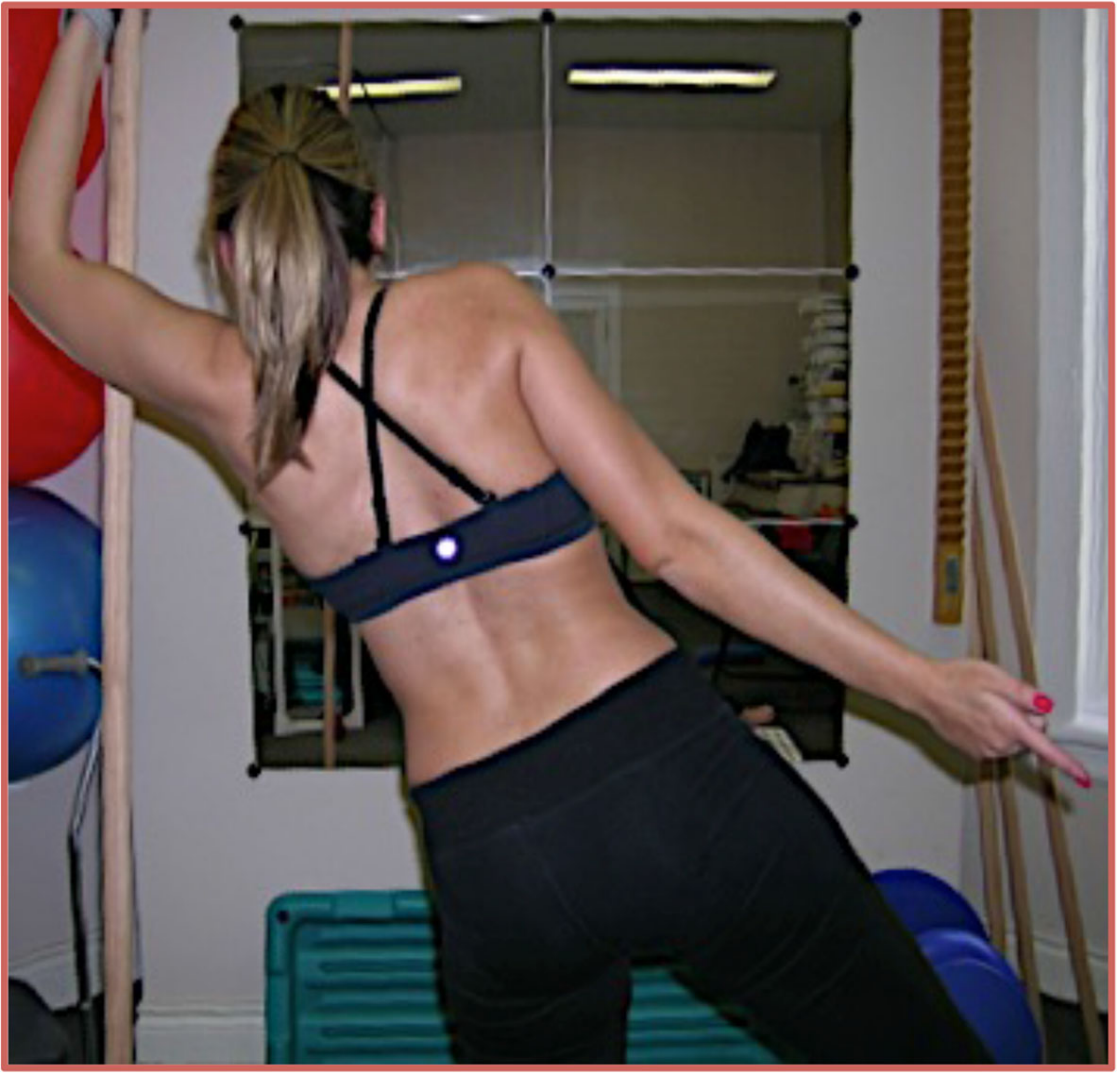
Photograph of the case presentation patient taken while performing Schroth physiotherapy exercises in July 2009. This specific exercise, “muscle cylinder,” promotes axial active self-elongation and 3D posture correction while focusing on rotational breathing techniques, directing inspired air into the concavities of the rib cage while maintaining the corrected posture

**Fig. 4:**
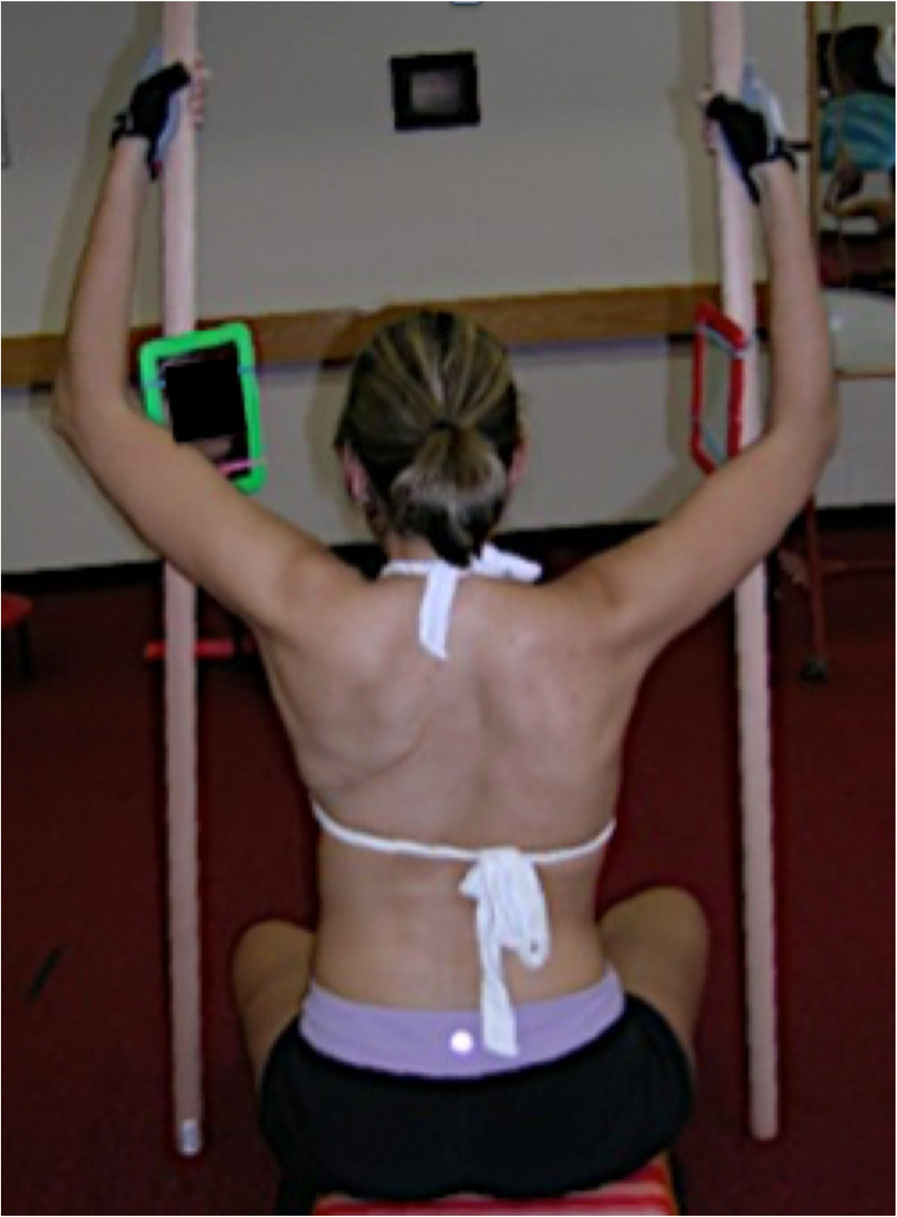
Photograph of the case presentation patient showing a corrected sitting posture while performing the “50 per” Schroth physiotherapy exercise in July 2009. This specific exercise promotes active self-elongation, right angular breathing techniques directing inspired air into the concavities of the rib cage while maintaining the corrected posture

Within 1 month of beginning Schroth physiotherapy (Figs. 3 and 4), the patient reported no more back pain and within 2 months reported improved breathing. In January 2009, physiotherapy visits were decreased to 2–4 times a month while the patient continued with a 45–60 min HEP 3–5 times a week. Over the next several months, air entry into the left lung improved and chest expansion increased from 1.5 cm to 4.0 cm from November 2008 to May 2009.

In July 2009, 9 months after beginning Schroth physiotherapy, radiographs showed reduced thoracic and lumbar Cobb angles of 58 and 43°, respectively (Fig. 2b). Private radiographs arranged by the patient taken during a Schroth exercise showed thoracic and lumbar Cobb angles of 48 and 33°, respectively (Fig. 2c). The patient showed continued improvement in vital capacity and ATR. She became more active and resumed all athletic activity by the end of summer 2009.

In November 2009, in preparation for rib separation surgery, the patient obtained a plastic Thoraco-lumbo-sacral-orthosis (TLSO) brace and an in-brace radiograph showed even further reductions in scoliosis curves to 38° thoracic Cobb angle and to 30° lumbar Cobb angle (Fig. 2d). Ultimately, the patient did not end up having the rib separation surgery and did not wear the TLSO brace that was made for her.

Since 2009, she continues to improve with independent Schroth home physiotherapy exercises 2–3 times a week. Her vital capacity increased from <2,000 mL in July 2008 to 3,200 mL in December 2014. She is now a mother of two, enjoys running marathons and mountain climbing, and is able to scuba dive in deep waters, surfacing with plenty of reserve oxygen supply. Her SRS-22 scores have been consistently high, ranging in >90^th^ percentile with scores of 4.50 (2011), 4.68 (2013), and 4.64 (2015).

## Conclusions

Adult scoliosis patients are not routinely referred for PSSE in Canada, even though Schroth physiotherapy, a form of PSSE, is shown to be effective in this case presentation. This case presentation patient was successfully treated with Schroth physiotherapy [23]. With an average of 1° annual progression for adults with scoliosis, the 12-degree reduction in Cobb angles from 70° to 58° for this patient saved her 12 years of progression in adulthood [18]. Long-term comprehensive Schroth physiotherapy, to help correct and maintain proper posture in all aspects of daily living, should be part of scoliosis management for adult scoliosis patients in Canada to stop and reverse curve progression. Furthermore, Schroth physiotherapy can help prevent and treat respiratory dysfunction [13] and spinal pain syndromes, prevent spinal fusion surgery, and improve vital capacity, spinal stability, aesthetics, postural appearance, and overall quality of life [24].

## Abbreviations

3D: Three-dimensional
AIS: Adolescent idiopathic scoliosis
ATR: Angle of trunk rotation
HEP: Home exercise program
IS: Idiopathic scoliosis
PSSE: Physiotherapeutic scoliosis-specific exercises
SRS: Scoliosis Research Society
SSSPT: Scoliosis specific Schroth physiotherapy
TLSO: Thoraco-lumbo-sacral-orthosis

## References

[1] Dubousset J. Three-dimensional analysis of the scoliotic deformity. In: The pediatric spine: principles and practice. New York: Raven Press Ltd; 1994. p. 479–96.

[2] Lonergan GJ, Schwab CM, Suarez ES, Carlson CL (2002). From the Archives of the AFIP: neuroblastoma, ganglioneuroblastoma, and ganglioneuroma: radiologic-pathologic correlation. RadioGraphics.

[3] Schreiber S, Parent EC, Watkins EM, Hedden DM (2012). An algorithm for determining scoliosis curve type according to Schroth. Scoliosis.

[4] Stokes IA, Burwell RG, Dangerfield PH (2006). IBSE: Biomechanical spinal growth modulation and progressive adolescent scoliosis--a test of the ‘vicious cycle’ pathogenetic hypothesis: summary of an electronic focus group debate of the IBSE. Scoliosis.

[5] Korovessis P, Papanastasiou D, Dimas A, Karayannis A (1993). Scoliosis by acquired rib fusion after thoracotomy in infancy. Eur Spine J.

[6] Sato T, Hirano T, Ito T, Morita O, Kikuchi R, Endo N (2011). Back pain in adolescents with idiopathic scoliosis: epidemiological study for 43,630 pupils in Niigata City, Japan. Eur Spine J.

[7] Pehrsson K, Bake B, Larsson S, Nachemson A (1991). Lung function in adult idiopathic scoliosis: a 20 year follow up. Thorax.

[8] Collis DK, Ponseti IV (1969). Long-term follow-up of patients with idiopathic scoliosis not treated surgically. J Bone Joint Surg Am.

[9] Marti CL, Glassman SD, Knott PT, Carreon LY, Hresko MT (2015). Scoliosis Research Society members attitudes towards physical therapy and physiotherapeutic scoliosis specific exercises for adolescent idiopathic scoliosis. Scoliosis.

[10] Bettany-Saltikov J, Parent E, Romano M, Villagrasa M, Negrini S (2014). Physiotherapeutic scoliosis-specific exercises for adolescents with idiopathic scoliosis. Eur J Phys Rehabil Med.

[11] Lehnert-Schroth C (1992). Introduction to the three-dimensional scoliosis treatment according to Schroth. Physiotherapy.

[12] Białek M, M'hango A, Kotwicki T (2007). Monitoring of changes in trunk rotation during scoliosis physiotherapy. Scoliosis.

[13] Weiss HR (1991). The effect of an exercise program on vital capacity and rib mobility in patients with idiopathic scoliosis. Spine (Phila Pa 1976).

[14] Huh S, Eun LY, Kim NK, Jung JW, Choi JY, Kim HS (2015). Cardiopulmonary function and scoliosis severity in idiopathic scoliosis children. Korean J Pediatr.

[15] Schroth C (2007). Three-dimensional treatment for scoliosis: Physiotherapeutic method for deformities of the spine.

[16] Fortin C, Feldman DE, Cheriet F, Gravel D, Gauthier F, Labelle H (2012). Reliability of a quantitative clinical posture assessment tool among persons with idiopathic scoliosis. Physiotherapy.

[17] Asher M, Min Lai S, Burton D, Manna B. The reliability and concurrent validity of the scoliosis research society-22 patient questionnaire for idiopathic scoliosis. Spine (Phila Pa 1976). 28(1):63–9. 10.1097/00007632-200301010-00015 12544958

[18] Negrini A, Parzini S, Negrini MG, Romano M, Atanasio S, Zaina F, Negrini S (2008). Adult scoliosis can be reduced through specific SEAS exercises: a case report. Scoliosis.

[19] Negrini S, Grivas TB, Kotwicki T, Maruyama T, Rigo M, Weiss HR, Members of the SOSORT (2006). Why do we treat adolescent idiopathic scoliosis? What we want to obtain and to avoid for our patients. SOSORT 2005 Consensus paper. Scoliosis.

[20] Marty-Poumarat C, Scattin L, Marpeau M. Garreau de Loubresse C, Aegerter P. Natural history of progressive adult scoliosis. Spine (Phila Pa 1976). 2007;32(11):1227–34. 10.1097/01.brs.0000263328.89135.a6 17495780

[21] Edgar MA (1987). The natural history of unfused scoliosis. Orthopedics.

[22] Brooks W, Krupinski EA, Hawes MC (2009). Reversal of childhood idiopathic scoliosis in an adult, without surgery: a case report and literature review. Scoliosis.

[23] Romano M, Minozzi S, Zaina F, Saltikov JB, Chockalingam N, Kotwicki T, Hennes AM, Negrini S (2013). Exercises for adolescent idiopathic scoliosis: a Cochrane systematic review. Spine (Phila Pa 1976).

[24] Schreiber S, Parent EC, Moez EK, Hedden DM, Hill D, Moreau MJ, Lou E, Watkins EM, Southon SC (2015). The effect of Schroth exercises added to the standard of care on the quality of life and muscle endurance in adolescents with idiopathic scoliosis - an sssessor and statistician blinded randomized controlled trial: “SOSORT 2015 Award Winner”. Scoliosis.

